# Effects of Adolescent-Focused Integrated Social Protection on Depression: A Pragmatic Cluster-Randomized Controlled Trial of Tanzania’s Cash Plus Intervention

**DOI:** 10.1093/aje/kwac093

**Published:** 2022-05-17

**Authors:** Leah Prencipe, Tanja A J Houweling, Frank J van Lenthe, Lusajo Kajula, Tia Palermo, on behalf of the Tanzania Adolescent Cash Plus Evaluation Team

**Keywords:** adolescent mental health, Cash Plus, depression, integrated interventions, social protection, Tanzania

## Abstract

We assessed the impacts of Tanzania’s adolescent-focused Cash Plus intervention on depression. In this pragmatic cluster-randomized controlled trial, 130 villages were randomly allocated to an intervention or control arm (1:1). Youth aged 14–19 years living in households receiving governmental cash transfers were invited to participate. The intervention included an intensive period (a 12-session course) and an aftercare period (9 months of mentoring, productive grants, and strengthened health services). We examined intervention impacts on a depressive symptoms scale (10-item Center for Epidemiologic Studies Depression Scale score (range, 0–30)) and rates of depressive symptomatology (score ≥10 points on the scale), recorded at study baseline (April–June 2017), midline (May–July 2018), and endline (June–August 2019). Using intention-to-treat methodology, we employed logistic and generalized linear models to estimate effects for binary and continuous outcomes, respectively. Quantile regression was used to estimate effects across the scale. From 2,458 baseline participants, 941 intervention and 992 control adolescents were reinterviewed at both follow-ups. At endline, the intervention reduced the odds of depressive symptomatology (adjusted odds ratio = 0.67, 95% confidence interval: 0.52, 0.86), with an undetectable mean scale difference (risk difference = −0.36, 95% confidence interval: –0.84, 0.11). Quantile regression results demonstrated an intervention effect along the upper distribution of the scale. Integration of multisectoral initiatives within existing social protection systems shows potential to improve mental health among youth in low-resource settings.

## Abbreviations

aOR: adjusted odds ratio
CI: confidence interval
CES-D: Center for Epidemiologic Studies Depression Scale
CES-D-10: 10-item Center for Epidemiologic Studies Depression Scale
ICC: intraclass correlation coefficient
LOC: locus of control
PSSN: Productive Social Safety Net
QR: quantile regression
SRH: sexual and reproductive health

Depression, one of the most frequent adolescent mental health disorders (1), causes the largest burden of disease in this population globally (2). Children suffering from depression are more likely to engage in substance use and delinquent behaviors, to attempt suicide, and to face social and educational challenges (3). While depression typically emerges during mid-to-late adolescence (1), the associated burdens of poor mental health can persist through adulthood (4) and continue into future generations (5), meaning that early interventions can have long-lasting impacts. While populations in low- and middle-income countries are acutely vulnerable to poor mental health (6), a scarcity of capital, workers, and services in these countries, along with stigma surrounding mental illness, contributes to a lack of progress in the management of mental health disorders (7).

Given the links between economic deprivation and poor mental health (6), interventions targeting poverty-related outcomes may also improve well-being. In a recent systematic review and meta-analysis, Zimmerman et al. (8) found that while cash transfers may improve some mental health outcomes for children and young people, they had a null effect on depressive symptoms. Among studies carried out in Africa, impacts of cash transfers on depressive symptoms were largely heterogeneous, with differential effects by school enrollment (9), household wealth (10), and sex (11, 12).

Considering the positive impacts of cash transfers related to poverty, food insecurity, and education (13), policy-makers are looking to build on these successes by providing complementary services within existing social protection programs (14). These integrated interventions have the potential to help recipients better leverage their benefits, while simultaneously minimizing implementation costs related to start-up, targeting, and capacity to provide services through the use of existing infrastructure. Because social determinants of mental health involve complex, multidimensional factors (15), interventions which incorporate targeted services in combination with cash payments (i.e., “cash plus” programs) may be more effective than cash alone.

Several studies in Africa have examined adolescent-focused interventions which combine economic strengthening components, such as cash grants, microcredit, vocational training, and/or financial education, with sexual and reproductive health training, violence prevention, mentoring, and/or use of “safe spaces” (16–18). While these “bundled” programs have shown potential to facilitate safe and healthy transitions to adulthood, the results largely focus on livelihoods, experiences of violence, gender norms, and sexual and reproductive health (SRH), overlooking potential benefits for mental health. The only (to our knowledge) peer-reviewed article on impacts on mental health evaluated the Girl Empower Program in Liberia, an intervention which combined cash transfers, girls’ empowerment training, and mentoring (18). While there were no measurable effects on psychosocial well-being, the intervention targeted girls aged 13–14 years, making the results ungeneralizable to adolescents at large.

We aimed to examine the added effect of a government-implemented, adolescent-targeted “plus” intervention on depressive symptoms among adolescents receiving cash benefits. This intervention combines livelihood and life skills training with linkage to health services, using a capability-building approach (19). The livelihood component was adapted from a previously implemented “start your own business” curriculum (20), while the life skills and SRH curricula were developed by TAMASHA, a Tanzanian nongovernmental organization with experience implementing similar interventions (21). An overall evaluation report on this intervention (22) found many positive economic effects, including improved employment aspirations, increased likelihood of starting a business, and increased livestock-keeping. These benefits extended into other areas by improving gender-equitable attitudes, reducing violence perpetration and experiences of sexual violence, and decreasing the proportion of adolescents reporting clinically relevant levels of depressive symptoms. In this paper, we conduct an in-depth analysis of the impacts of the intervention on depressive symptoms and discuss them in the context of the broader literature.

## METHODS

### Study design and participants

**Figure 1:**
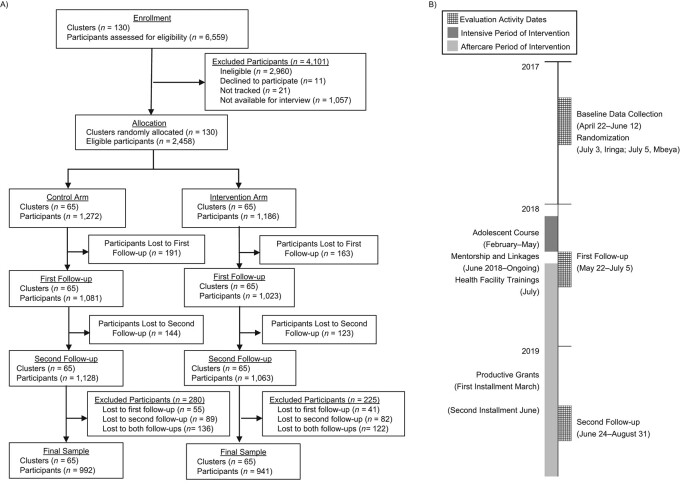
Profile (A) and timeline (B) of the Cash Plus intervention among adolescents in Tanzania, 2018–2019. The flow chart (A) shows the flow of clusters and participants through the trial, beginning with the enrollment period, continuing through randomization and 2 follow-up data collection periods, and concluding with the final analytical sample. The timeline (B) details the dates of key evaluation activities and gives an overview of intervention activities from prerandomization through the second follow-up data collection period.

This pragmatic cluster-randomized controlled trial (Figure 1) was conducted in 2 government administrative areas of mainland Tanzania, one within Iringa and the other in Mbeya, to measure the impacts of the intervention on youth well-being, violence reduction, and safe transitions to adulthood. Because the intervention was designed to be integrated into the existing systems and framework of the Productive Social Safety Net (PSSN), a large-scale governmental conditional cash transfer program, these areas were already providing cash benefits to eligible households prior to selection. From the 211 PSSN-enrolled villages within the study areas, we excluded villages that 1) were engaged in parallel PSSN evaluations or 2) had fewer than 10 or more than 100 adolescents aged 14–19 years according to PSSN-beneficiary listings. Local officials and community leaders from the remaining 130 villages agreed to participate in both the study and the intervention.

Eligible participants were aged 14–19 years and living in PSSN-beneficiary households in 2017. PSSN-beneficiary listings from 2015 were used to identify participants prior to recruitment (April 19–21, 2017). Largely due to changes in household composition since 2015, approximately 45% of potential respondents were ineligible because they were outside of the designated age range (*n* = 745), they were no longer living in PSSN-beneficiary households (*n* = 1,724), or the household respondent refused or did not consent (*n* = 491). We obtained ethical approval from the National Institute for Medical Research and the Tanzania Commission for Science and Technology. The trial was registered retrospectively in the Pan-African Clinical Trials Registry (trial PACTR201804003008116) on January 25, 2018.

### Randomization and blinding

During public randomization events (July 3, 2017, in Iringa; July 5, 2017, in Mbeya), 130 eligible villages were randomized at a 1:1 ratio to an intervention or control arm, stratified by region and number of eligible youths per village (less than the sample median vs. greater than or equal to the sample median). Blinding of participants and implementers was not possible due to the nature of the intervention, and survey components on fidelity of implementation precluded the blinding of enumerators.

### Procedures

The Ujana Salama (Swahili for “safe youth”) intervention utilizes a “cash plus” model, wherein synergies between direct financial support (cash) and complementary programming (plus) are used to address multifactoral risk factors for poor economic, social, and health outcomes. The initial 3-month intensive period of the intervention consisted of a 12-session course for adolescents. Employing a community-based approach, as opposed to clinic- or school-based (which may limit access), and to reflect recruitment in real-world settings, all youths aged 14–19 years living in PSSN-beneficiary households were invited to attend the course through community messaging mechanisms and at PSSN payment points.

**Figure 2:**
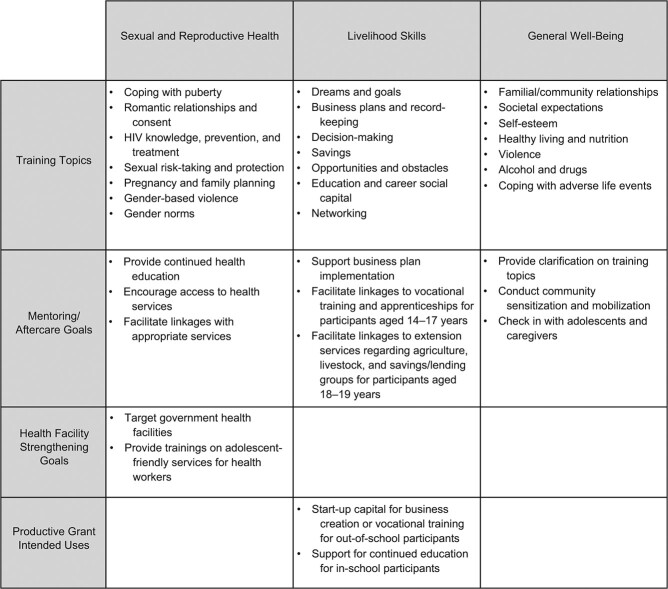
Overview of the Tanzania Adolescent Cash Plus Intervention according to main topic and component, Tanzania, 2018–2019. The figure shows the topics and objectives of each intervention component (adolescent training, mentoring/aftercare, health facility strengthening, and productive grants) as categorized by the main themes of the intervention (sexual and reproductive health, livelihood skills, and general well-being). HIV, human immunodeficiency virus.

Trained and supported by PSSN implementers, community instructors provided weekly sessions (2–4 hours each) aimed to increase skills and resources related to education, economic activities, SRH, and general well-being. Following the intensive period, adolescents who attended the course were offered aftercare services through mentoring, linkages to health services, and, for those who submitted a business or education plan, direct productive grants (approximately $80 US). To address supply-side barriers, adolescent-friendly trainings were conducted during the aftercare period among government health facilities serving intervention communities. Figure 2 provides an overview of the intervention components and topics covered, as summarized from the overall evaluation report (22).

Control clusters (*n* = 65) received no additional services during the study (delayed intervention started in 2021); however, household cash transfer benefits did not differ between study arms. While unlikely, it was possible for participants from control communities to access health facilities serving intervention communities.

Baseline data were collected between April 22 and June 12, 2017, with plans to measure impacts 6 and 18 months after the intensive intervention period. Because of programmatic delays, the first follow-up (study midline: May 22–July 5, 2018) was conducted immediately after completion of the 12-session course (0 months after the intensive period). The second follow-up (study endline: June 24–August 31, 2019) commenced after 9 months of mentoring activities and 1–2 months after final grant disbursements (12 months after the intensive period). Data collection was not rescheduled to accommodate intervention delays, since many study outcomes, including livelihood activities, are sensitive to seasonality in the study areas. Surveys were translated to Swahili and pilot-tested. Written informed consent was obtained from participants aged 18 years or older and married youth of any age; otherwise, written consent was obtained from guardians, in addition to verbal assent from minors.

### Outcomes

We used the 10-item version (CES-D-10) of the Center for Epidemiologic Studies Depression Scale (CES-D) (23) to estimate the effects of the intervention on mental health. The CES-D-10, a short form of the 20-item CES-D, measures severity of depressive symptomology and has been validated among adolescents in Tanzania and other African countries (24). Participants’ responses to 10 questions on feelings and behaviors during the prior 7 days ranged from 0 (rarely; 0–1 days) to 3 (all of the time; 6–7 days) (see Web Table 1, available at https://doi.org/10.1093/aje/kwac093, for psychosocial survey items). Three items measuring positive affect were reverse-coded, and subsequently all responses were summed to create a depressive symptoms scale (range, 0–30 points), wherein higher scores reflect higher depression severity. Although not a diagnostic tool, a threshold score of 10 or more points on the CES-D-10 has been used in similar populations (24) and was used here to indicate youth exhibiting depressive symptomatology. The tandem use of categorical and dimensional outcomes of depressive symptoms provides complementary evidence.

The secondary goal of this study was to acquire a mechanistic understanding of impacts on mental health by estimating effects on potential pathways. We selected time-variant indicators, aligned with the intervention curriculum, topics, or goals, that were identified in a previous study as important factors related to depressive symptoms in the baseline sample of this evaluation (25): employment/education status, having a romantic partner, self-esteem, quality of life, and locus of control (LOC).

To measure youth employment and education status, youths were asked whether they were attending school or any training programs (including vocational training) and about paid employment outside of the household during the prior 7 days. We created the following 4 categorical indicators: in school or training but not in paid work; in paid work but not in school or training; in both paid work and school or training; and not in employment, education, or training. Youth were classified as having a romantic partner if they were married, were cohabitating, or reported having a girlfriend or boyfriend. Self-esteem was measured using the mean score (1–5 points) of 2 Rosenberg’s Self-Esteem Scale (26) items. Self-perceived quality of life was measured using a 10-point scale (27). LOC, a construct designed to measure whether control over one’s life outcomes is mostly internal (a person has control over one’s own life) or external (life is controlled by outside factors), was measured using the mean score (1–5 points) of 5 items from Levenson’s multidimensional LOC scale (28). A higher LOC score indicates more internal control. All outcomes were measured at baseline and both follow-ups.

### Statistical analysis

We used a sample size calculation to determine the number of clusters required. Because mental health was a secondary outcome for the overall evaluation, the calculation was based on the primary outcomes: pregnancy, transactional sex, one’s first experience of sex being forced, physical violence, and violence reporting. We estimated intraclass correlation coefficients (ICCs) of 0–0.14 based on data from a Tanzanian study carried out among adolescents and youth in similar households, and we note that many of these outcomes measure low–base-rate behaviors (i.e., contain many zeros) in any pragmatically recruited sample. Using Stata’s (StataCorp LLC, College Station, Texas) power and sample size commands (“sampsi”), 65 clusters with 9–18 adolescents each were required for a minimum detectable effect size of a 5–percentage-point change for binary outcomes with a power of 0.80 (2-tailed *P* < 0.05).

Analyses used intention to treat, including all clusters and adolescents, irrespective of intervention uptake or whether youth remained living in PSSN-beneficiary households at follow-up (cash component). Intervention effects of the adolescent-focused components (over cash alone) were estimated at midline and endline using linear mixed models with village-level random effects to account for clustering at the village level. The regression models adjusted for age, sex, the baseline values of outcomes, and strata (region and village size). For continuous outcomes, standardized effect sizes are reported, and for binary outcomes, adjusted odds ratios (aORs) are reported. The sample included all adolescents (*n* = 1,933) with baseline, midline, and endline data.

We also examined the distribution of CES-D-10 scores using data visualization, as positive skewness for the original and modified versions of the CES-D (29) is common in nonpsychiatric populations. Given the skewed distribution of the data, in addition to the mean effect provided by linear mixed models, a quantile regression (QR) approach was used to estimate effects across the scale. QR estimates utilized matched parameters (adjustments for youth characteristics, village clustering, and stratification) of the previous analyses and were represented graphically. The QR approach not only provides a more comprehensive picture of effects across levels, particularly for outcomes with nonnormal distributions, it also reduces the Type 1 error related to heteroscedasticity, making no assumptions about the distribution of the residuals (30). Data management and analysis were performed using Stata, version 16.1. Additional visualizations were performed using R, version 4.0.2 (R Foundation for Statistical Computing, Vienna, Austria).

## RESULTS

Between April and June 2017, a total of 6,559 individuals identified via PSSN-beneficiary listings were screened for eligibility, which resulted in 2,458 interviewed adolescents at baseline (Figure 1). All 130 eligible clusters were randomized equally to the intervention (*n* = 1,186) and control (*n* = 1,272) arms. A total of 1,933 adolescents were reinterviewed at both follow-ups (79%), with no difference between the intervention (79%) and control (78%) samples (*P =* 0.163).

Table 1 shows that 868 girls (45%) and 1,065 boys (55%) were included in the analysis. On average, participants were 16 years old, with approximately two-thirds (66%) living in female-headed households. Household characteristics were similar between study arms, although the intervention group had lower wealth levels (*P* < 0.001). Most youths were either exclusively in school (53%) or not in employment, education, or training (32%), with just 15% engaging in paid work (11% exclusively; 4% were in both school and paid work). Youth in the control group were more likely to have a romantic partner (19%) than those in the intervention group (15%) (*P =* 0.009). On average, youth had a depression score of 6.66, with 554 adolescents (29%) exhibiting clinically relevant levels of depressive symptomatology; there were no differences between study arms.

**Table 1:** Baseline Characteristics of Participants in an Evaluation of the Effect of a Cash Plus Intervention on Adolescent Depression, by Intervention Group, Tanzania, 2018–2019

	**Total** **(*n* = 1,933)**		**Intervention Group** **(*n* = 941)**		**Control Group** **(*n* = 992)**		***P* Value** ^**a**^
**Characteristic**	**No.**	**%**	**No.**	**%**	**No.**	**%**	
Household characteristics							
Wealth level (asset-based index)^b^							<0.001
Poorest third	632	33	357	38	275	28	
Middle third	655	34	316	34	339	34	
Richest third	644	33	266	28	378	38	
Household size, no. of persons^c^	4.97 (1.98)		5.00 (1.99)		4.94 (1.98)		0.480
Female-headed household (yes)	1,275	66	609	65	666	67	0.262
City							0.950
Iringa	971	50	472	50	499	50	
Mbeya	962	50	469	50	493	50	
Adolescent characteristics							
Sex							0.251
Female	868	45	410	44	458	46	
Male	1,065	55	531	56	534	54	
Age, years^c^	16.04 (1.59)		16.00 (1.55)		16.08 (1.63)		0.297
Education/employment status							0.291
Attending school/in training	1,019	53	498	53	521	53	
Engaged in paid work	218	11	95	10	123	12	
In both school/training and paid work	74	4	33	4	41	4	
Not in employment, education, or training	622	32	315	33	307	31	
Having a romantic partner (yes)	328	17	138	15	190	19	0.009
Social support score (range, 1–5)^c^^,^^d^	3.99 (0.62)		4.02 (0.63)		3.97 (0.61)		0.152
Adolescent psychosocial well-being							
Self-esteem score (range, 1–5)^c^^,^^e^	3.95 (0.77)		3.97 (0.77)		3.93 (0.78)		0.300
Quality of life score (range, 1–10)^c^^,^^f^	3.83 (2.35)		3.89 (2.48)		3.76 (2.22)		0.219
Locus of control score (range, 1–5)^c^^,^^g^	3.20 (0.48)		3.20 (0.48)		3.20 (0.47)		0.841
Adolescent mental health							
Depression scale score (range, 1–30)^c^^,^^h^	6.66 (4.88)		6.67 (4.76)		6.65 (5.00)		0.916
Depressive symptomatology (yes)^i^	554	29	274	29	280	28	0.665

Abbreviation: CES-D-10, 10-item Center for Epidemiologic Studies Depression Scale.

^a^ *P* values were derived from an independent *t* test for continuous variables and a χ^2^ test for categorical variables.

^b^ Information on household wealth was not available for 2 observations.

^c^ Values are expressed as mean (standard deviation).

^d^ Social support was measured using the average of 4 Multidimensional Scale of Perceived Social Support (31) items.

^e^ Self-esteem was measured using the average of 2 Rosenberg’s Self-Esteem Scale (26) items.

^f^ Quality of life was measured using the 10-point Cantril’s Ladder of Life Scale (27).

^g^ Locus of control score was measured using the average of 5 Levenson’s Multidimensional Locus of Control Scale (28) items.

^h^ Depressive symptoms were measured using the CES-D-10 scale.

^i^ Depressive symptomatology was defined as a score of ≥10 points on the CES-D-10 scale.

Baseline characteristics of the intervention group are provided in Web Table 2 by intervention uptake. While half (50%) of the intervention group attended at least 1 session of the adolescent course, less than one-third met with a mentor (28%) or received a grant (30%). The characteristics of youth who attended at least 1 session of the course (*n* = 475) were mostly similar to those of youth who did not (*n* = 466), apart from region, sex, and LOC. The youths who attended were more likely to be from Mbeya, to be female, and to have a higher internal LOC, compared with the nonparticipatory group. Youths who engaged in mentorship (*n* = 258) and grant procurement (*n* = 278) activities were also more likely to be female and to live in Mbeya than those who did not, but no discernible difference was found for LOC. Intervention youth who received a productive grant reported slightly lower levels of social support (31) than those who did not.

### Intervention effect

We measured effects on primary and exploratory outcomes (Table 2) immediately after the intensive intervention period (midline) and again 12 months later (endline), following productive grant distribution, training of health-care providers, and at least 9 months of mentoring.

**Table 2:** Effects of a Cash Plus Intervention on Primary and Exploratory (Mediating) Outcomes Among Adolescents at Study Midline and Endline, by Intervention Group, Tanzania, 2018–2019

	**Intervention Group** **(*n* = 941)**		**Control Group** **(*n* = 992)**		**Risk Difference** ^**b**^	**95% CI** ^**c**^	**Odds Ratio** ^**b**^	**95% CI** ^**c**^	***P* Value**	**ICC**
**Outcome** ^**a**^	**No.**	**%**	**No.**	**%**						
*Midline*										
Primary outcomes										
Depression scale score (range, 1–30)^d^	6.88 (4.02)		6.67 (4.08)		0.15	−0.30, 0.60			0.506	0.03
Depressive symptomatology (yes)	232	25	252	25			0.93	0.70, 1.23	0.617	0.06
Exploratory outcomes										
Education/employment status										
Attending school/in training	410	44	446	45			0.87	0.66, 1.14	0.304	0.03
Engaged in paid work	96	10	100	10			1.07	0.70, 1.61	0.764	0.12
In both school/training and paid work	42	4	41	4			1.14	0.69, 1.89	0.613	0.10
Not in employment, education, or training	393	42	405	41			1.00	0.80, 1.26	0.975	0.02
Having a romantic partner (yes)	305	32	350	35			0.94	0.72, 1.23	0.658	0.05
Self-esteem score (range, 1–5)^d^^,^^g^	3.77 (0.78)		3.76 (0.78)		0.00	−0.10, 0.09			0.922	0.04
Quality of life score (range, 1–10)^d^^,^^h^	5.26 (2.67)		5.12 (2.56)		0.06	−0.30, 0.43			0.742	0.09
Locus of control score (range, 1–5)^d^^,^^i^	3.30 (0.44)		3.29 (0.44)		0.01	−0.03, 0.05			0.469	0.00
*Endline*										
Primary outcomes										
Depression scale score (range, 1–30)^d^	5.62 (4.33)		6.03 (4.78)		−0.36	−0.84, 0.11			0.134	0.02
Depressive symptomatology (yes)	182	19	262	26			0.67	0.52, 0.86	0.001	0.02
Exploratory outcomes										
Education/employment status										
Attending school/intraining	295	31	328	33			0.88	0.67, 1.14	0.326	0.02
Engaged in paid work	219	23	203	20			1.28	1.00, 1.64	0.049	0.01
In school/training and paid work	21	2	44	4			0.46	0.24, 0.87	0.017	0.18
Not in employment, education, or training	406	43	417	42			1.02	0.82, 1.28	0.854	0.02
Having a romantic partner (yes)	391	42	409	41			1.09	0.87, 1.36	0.452	0.02
Self-esteem score (range, 1–5)^d^^,^^g^	3.86 (0.80)		3.76 (0.81)		0.10	0.03, 0.18			0.007	0.01
Quality of life score (range, 1–10)^d^^,^^h^	4.85 (2.12)		4.80 (1.99)		0.03	−0.20, 0.26			0.801	0.03
Locus of control score (range, 1–5)^d^^,^^i^	3.29 (0.46)		3.28 (0.47)		0.01	−0.03, 0.05			0.549	0.00

Abbreviations: CES-D-10, 10-item Center for Epidemiologic Studies Depression Scale; CI, confidence interval; ICC, intraclass correlation coefficient.

^a^ Primary outcomes were measured using the CES-D-10, as well as a binary measurement (CES-D-10 score ≥10 points) that indicated depressive symptomatology.

^b^ Adjusted for sex, age (years), the corresponding outcome at baseline, and district/community size fixed effects (the strata). Multilevel methodology was used to account for clustering of outcomes within and between villages.

^c^ Robust 95% CIs.

^d^ Values are expressed as mean (standard deviation).

^e^ Depressive symptoms were measured using the CES-D-10 scale.

^f^ Depressive symptomatology was defined as a score of ≥10 points on the CES-D-10 scale.

^g^ Self-esteem score was the average of 2 Rosenberg’s Self-Esteem Scale (26) items.

^h^ Quality of life was measured using a 10-point Cantril’s Ladder of Life Scale (27).

^i^ Locus of control score was the average of 5 Levenson’s Multidimensional Locus of Control Scale (28) items.

Immediately following the intensive period, there were no intervention effects found for depression scale score (risk difference = 0.15, 95% confidence interval (CI): –0.30, 0.60), for the prevalence of depressive symptomatology (CES-D-10 score ≥10) (aOR = 0.93, 95% CI: 0.70, 1.23), or for any potential pathway indicators. Twelve months later, the intervention had reduced the odds of having depressive symptomatology by 33% (aOR = 0.67, 95% CI: 0.52, 0.86), with no detectable difference in the mean number of symptoms (risk difference = −0.36, 95% CI: –0.84, 0.11). Results were consistent by sex (Web Table 3). At endline, we also found reduced odds for youth to be engaged in both paid work and school (aOR = 0.46, 95% CI: 0.24, 0.87) and increased odds of exclusive paid work (aOR = 1.28, 95% CI: 1.00, 1.64). There were also modest gains in self-esteem (risk difference = 0.10, 95% CI: 0.03, 0.18) at endline.

**Figure 3:**
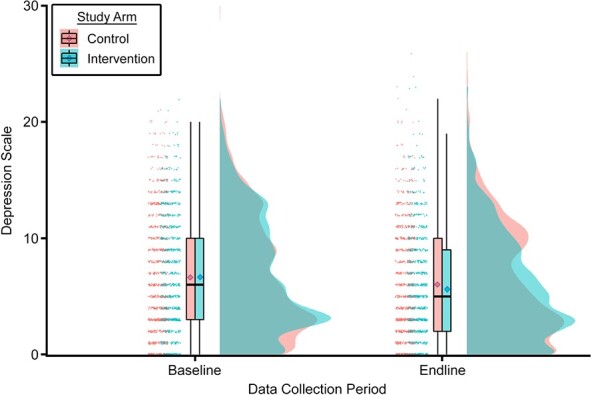
Distribution of depressive symptoms by intervention status at baseline and endline in the Tanzania Adolescent Cash Plus Evaluation, Tanzania, 2018–2019. Depressive symptoms were measured using the 10-item Center for Epidemiologic Studies Depression Scale. Group distributions are depicted using raincloud plots: individual data points (horizontally jittered), box plots with 95% confidence intervals, and unmirrored moderately smoothed violin plots (probability density functions). In the box plots, the borders of the boxes represent the 25th (quartile 1) and 75th (quartile 3) percentiles (i.e., the interquartile range (IQR)); the horizontal line inside the box represents the median value; and the diamonds represent the mean score for each group. The whiskers extend to the minimum (without outliers) and maximum (without outliers) values, as calculated by quartile 1/quartile 3 ± 1.5 × IQR. Outliers are not shown in the box plots but can be seen in the horizontally jittered data points.

Individual rain-cloud plots by study arm show the distribution (cloud) of observations (rain drops) for depressive symptoms at both baseline and endline (Figure 3). While there were no discernable differences in distribution at baseline between the intervention and control groups, at endline the box plots showed similar distributions for the first and second quartiles (25th percentile = 2; 50th percentile = 5), with differences emerging at the higher end of the distribution (control 75th percentile = 10; intervention 75th percentile = 9).

**Figure 4:**
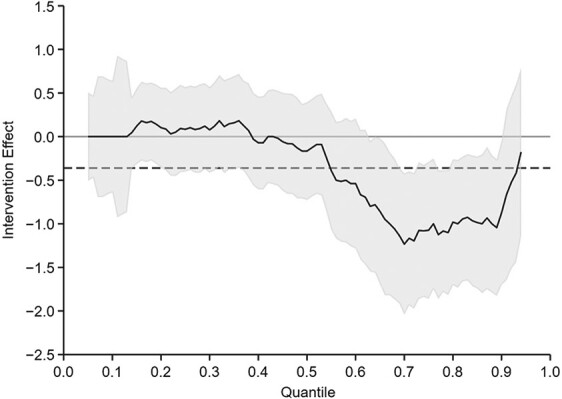
Results of quantile regression analysis of depressive symptoms at endline in the Tanzania Adolescent Cash Plus Evaluation, Tanzania, 2018–2019. Depressive symptoms were measured using the 10-item Center for Epidemiologic Studies Depression Scale. The solid line represents the effect of the intervention along the quantile distribution, and the gray shading indicates the 95% confidence interval. The dashed line shows the effect on the mean value (not significant; no confidence interval shown). Estimates were adjusted for age, sex, baseline score, and sampling strata. Standard errors were clustered at the village level.

Figure 4 displays the QR-estimated intervention effects from the fifth to the 95th percentiles of the depression scale. The QR showed that depressive symptoms were lower in the intervention group than in the control group between the 65th and 90th percentiles of the distribution. For example, at the 75th percentile, the intervention group had a 1.07-point lower depression score (95% CI: –1.86, −0.29) than the control group.

## DISCUSSION

This is the first known randomized controlled trial of a government-implemented, integrated social protection program targeting adolescents in Africa. While we found no impacts immediately after the intensive intervention period, the proportion of participants exhibiting depressive symptomatology was reduced 12 months later, after additional intervention components had been implemented. Potential mechanisms for reduced symptomatology include decreased engagement in paid work while attending school and improved self-esteem. Despite no overall impact on the depressive symptoms scale, fewer symptoms were found among the top percentiles of the distribution. This underscores that the intervention was most successful in reducing depressive symptoms among adolescents who were most at risk.

Although traditional statistical models base estimates on the mean value of the dependent variable, this is not always a robust measure, particularly when data are highly skewed (32) like those on our CES-D-10 scale, and it may overlook differential effects across the distribution or around important clinical thresholds. As demonstrated here, the linear mixed model gave equal weight to effects along the scale, resulting in null effects. Because the intervention reduced the odds of depressive symptomatology, we concluded that the mean difference in the scale score was not sensitive enough to detect a shift in the distribution between study arms.

The results of the QR can be further interpreted using the visualization of the depressive symptom scale at endline (Figure 3), wherein control participants exhibited symptoms in a bimodal distribution (i.e., “heaped” around 2 values) as opposed to the unimodal distribution of the intervention group, noted by a single peak, followed by a steady downward slope. The baseline depression scale also peaks around the low end of the distribution (no depressive affect) but, similar to the control endline group, increases again around the depressive symptomatology threshold. While few published papers display the distribution of CES-D scores, the bimodal distribution has been previously observed in nonclinical populations (33, 34). The apparent grouping of nondepressed and depressed individuals may further justify the use of thresholds to determine risk, particularly among community samples, and confirms the need for methodology which accommodates nonnormal distributions.

Low self-esteem (as compared with high self-esteem) and engaging in both school and paid work (as compared with exclusive schooling) were associated with poorer mental health at baseline (25), and our study found modest protective intervention effects on these pathways. However, the pathway indicators examined here do not fully explain the effects on mental health. In work published elsewhere, investigators found that the intervention increased equitable gender attitudes (35) and reduced experiences of sexual violence and the perpetration of physical violence (36). These results may have contributed to better mental health, as violence perpetration among males is associated with increased substance use, less gender-equitable attitudes, and posttraumatic stress disorder and depression (37). Furthermore, experiencing sexual violence in childhood increases the likelihood of developing mental health disorders (38).

Considering the links between economic status and mental health (6), outcomes related to economic empowerment may have mediated the impacts on mental health. In the overall evaluation, study investigators reported that by endline, the intervention led to more adolescents starting and investing in their own businesses and participating in livestock-keeping activities (22). Additionally, they reported a decrease in school attendance attributable to the intervention. Adding to these findings, while our study did not find negative effects on exclusive schooling, the percentage of participants engaged in both schooling and paid work decreased. We posit that when presented with the choice to continue their education along with the additional burden of paid work, intervention youth were more inclined to focus solely on income-generating activities than youth in control communities. The relatively high ICC at endline for being in both school and paid work (ICC = 0.18), as compared with other education/employment statuses (ICC range, 0.01–0.02), indicates that where these youth lived was also important. Because this study included a highly vulnerable population, investments in productive activities may have increased hope and resilience among youth with few formal economic prospects, further influenced by community factors such as availability and access to secondary schools. When designing livelihood interventions across the highly transformative period of late adolescence, strengthening secondary schools and reducing barriers to quality education might help alleviate this unintended outcome.

We hypothesize that unmeasured mechanisms related to mentorship may have mediated the mental health effects. Because mentors were selected from the communities in which these youth lived, we hypothesize that the intervention increased levels of social capital and community cohesion, which are both important predictors of mental health among adolescents (39). Moreover, null results immediately after the training (i.e., at midline) suggest that livelihood and life-skills trainings alone do not impact mental health, or that more time was needed for changes to take effect. Aligned with the intervention’s multidimensional conceptualization of determinants of well-being, the results suggest that it is precisely the multisectoral implementation, addressing various capabilities, that ultimately had synergistic, protective effects on mental health.

Because this intervention incorporates SRH and livelihood-strengthening components on top of cash transfer benefits, our study adds to the current literature in several ways. A recent systematic review of SRH interventions highlighted the paucity of evidence related to psychosocial and mental health outcomes, despite the strong associations between poor mental health and negative SRH outcomes, particularly among women (40). Similarly, little is known regarding the mental health benefits of livelihood interventions, which focus on building skills over time (41).

A previous study of the PSSN, in which both intervention and control youth from this study were enrolled, found adverse effects of the conditional cash transfer among females (12). The authors postulated that program conditions, wherein a base monthly household cash transfer (approximately $5–$7 US) increased as much as 3 times (maximum approximately $18 US), contingent on compliance with program requirements, may have contributed to worse mental health among women. Conditional obligations, such as taking children for health checkups and ensuring compliance for school attendance, were mostly borne by women, regardless of recipient status, placing the increased time burdens for this additional income on individuals who were already overloaded with domestic duties. Although we might have expected enrollment in the PSSN to influence the effect of the current intervention among girls, the women who experienced adverse effects on mental health in the PSSN study were older and more likely to be pregnant before and during the study period, and thus were more likely to be affected by the hidden, gendered costs associated with meeting program conditions than youth in our current study.

There were some limitations to this study. First, while the intervention was implemented as a Cash Plus model, the results of our study represent only the added effect of the “plus” and not the effects of the cash alone or the synergies between the 2 elements. The adolescent-focused components (the “plus”) were added on top of the PSSN cash transfer benefits (the “cash”), which were distributed to both study arms starting 2 years before baseline. Secondly, delays in implementing the adolescent training and disruptions in PSSN payments during the study period (May 2019–July 2020) may have mitigated potential impacts of the program. Finally, due to the lack of separate arms for plus components, the shorter duration of time between implementation and follow-up periods, and the overlap in uptake for the intensive and aftercare components, as per the intervention design, it was not possible to disentangle effects from the separate plus components. However, because the intervention was implemented within the existing framework of a program reaching over 1.1 million impoverished households, these results are meaningful for policy-makers in Tanzania and in other low-resource settings.

In conclusion, our results suggest that the integration of multisectoral initiatives within existing governmental social protection systems can provide secondary benefits among adolescents by reducing depressive symptomatology. In future research, investigators should explore pathways of effects related to community-based mentorship.

## Supplementary Material

Web_Material_kwac093
