# Supplementary material for: Effects of Adolescent-Focused Integrated Social Protection on Depression: A Pragmatic Cluster-Randomized Controlled Trial of Tanzania’s Cash Plus Intervention
Source: Am J Epidemiol. 2022 May 17;191(9):1601–13. doi: 10.1093/aje/kwac093 (PMC9437816; doi:10.1093/aje/kwac093)
Supplement: Web_Material_kwac093 [file web_material_kwac093.pdf]

WEB MATERIAL

Effects of Adolescent-Focused Integrated Social Protection on Depression: A Pragmatic Cluster-Randomized Controlled Trial of Tanzania's Cash Plus Intervention

Leah Prencipe, Tanja AJ Houweling, Frank van Lenthe, Lusajo Kajula, and Tia Palermo on behalf of the Adolescent Cash Plus Evaluation Team

Table of Contents

Web Table 1 2
Web Table 2 4
Web Table 3 5

Web Table 1. Psychosocial survey items

10-item Center for Epidemiologic Studies Depression Scale
(1) In the past seven days, how often did you sleep well? (Reverse-coded)<br>*0 = rarely (< 1 day)*<br>*1 = some or a little of the time (1–2 days)*<br>*2 = occasionally or a moderate amount of time (3–4 days)*<br>*3 = most or all of the time (5–7 days)*<br><br>*(2) In the past seven days, how often were you happy? (Reverse-coded)*<br>*0 = rarely (< 1 day)*<br>*1 = some or a little of the time (1–2 days)*<br>*2 = occasionally or a moderate amount of time (3–4 days)*<br>*3 = most or all of the time (5–7 days)*<br><br>*(3) In the past seven days, how often did you have trouble concentrating?*<br>*0 = rarely (< 1 day)*<br>*1 = some or a little of the time (1–2 days)*<br>*2 = occasionally or a moderate amount of time (3–4 days)*<br>*3 = most or all of the time (5–7 days)*<br><br>*(4) In the past seven days, how often did you feel hopeful about the future? (Reverse-coded)*<br>*0 = rarely (< 1 day)*<br>*1 = some or a little of the time (1–2 days)*<br>*2 = occasionally or a moderate amount of time (3–4 days)*<br>*3 = most or all of the time (5–7 days)*<br><br>*(5) In the past seven days, how often did you feel that everything you did was an effort?*<br>*0 = rarely (< 1 day)*<br>*1 = some or a little of the time (1–2 days)*<br>*2 = occasionally or a moderate amount of time (3–4 days)*<br>*3 = most or all of the time (5–7 days)*<br><br>*(6) In the past seven days, how often did you feel lonely?*<br>*0 = rarely (< 1 day)*<br>*1 = some or a little of the time (1–2 days)*<br>*2 = occasionally or a moderate amount of time (3–4 days)*<br>*3 = most or all of the time (5–7 days)*<br><br>*(7) In the past seven days, how often did you feel depressed?*<br>*0 = rarely (< 1 day)*<br>*1 = some or a little of the time (1–2 days)*<br>*2 = occasionally or a moderate amount of time (3–4 days)*<br>*3 = most or all of the time (5–7 days)*<br><br>*(8) In the past seven days, how often did you feel that you could not get going?*<br>*0 = rarely (< 1 day)*<br>*1 = some or a little of the time (1–2 days)*<br>*2 = occasionally or a moderate amount of time (3–4 days)*<br>*3 = most or all of the time (5–7 days)*<br><br>*(9) In the past seven days, how often were you bothered by things that don't usually bother you?*<br>*0 = rarely (< 1 day)*<br>*1 = some or a little of the time (1–2 days)*<br>*2 = occasionally or a moderate amount of time (3–4 days)*<br>*3 = most or all of the time (5–7 days)*<br><br>*(10) In the past seven days, how often did you feel fearful?*<br>*0 = rarely (< 1 day)*<br>*1 = some or a little of the time (1–2 days)*<br>*2 = occasionally or a moderate amount of time (3–4 days)*<br>*3 = most or all of the time (5–7 days)*

Self-esteem
How do you see yourself relative to the following statements? Please rate from 1 (strongly agree) to 5 (strongly disagree).<br>*(1) You feel that you have many good qualities.*<br>*(2) On the whole, you are satisfied with yourself.*
Quality of life
Imagine a ladder where on the bottom, the first step, represents the worst possible life for you and the highest step, the tenth, represents the best possible life for you. On which step of the ladder would you say you are today?
Locus of control
Please rate from 1 (strongly agree) to 5 (strongly disagree).<br>*(1) It's not always wise for you to plan too far ahead because many things turn out to be a matter of good or bad fortune (reverse-coded)*<br>*(2) Your life is determined by your own actions*<br>*(3) When you get what you want, it is usually the result of your own actions*<br>*(4) You feel like what happens in your life is mostly determined by others (reverse-coded)*<br>*(5) Getting what you want requires pleasing the influential people (reverse-coded)*

Web Table 2. Baseline Characteristics of Participants in the Intervention Group by Intervention Uptake, Adolescent Cash Plus Evaluation, Tanzania, 2018–2019

Characteristic	Attended Course			Met With Mentor [a]			Received Productive Grant		
	Yes (n=475)	No (n = 466)		Yes (n=258)	No (n=664)		Yes (n=278)	No (n=663)	
	No. (%)	No. (%)	*P* value [b]	No. (%)	No. (%)	*P* value [b]	No. (%)	No. (%)	*P* value [b]
**Household characteristics**									
Wealth level [c]			0.533			0.773			0.129
Poorest	183 (39%)	174 (38%)		94 (36%)	258 (39%)		113 (41%)	244 (37%)	
Middle	165 (35%)	151 (33%)		89 (34%)	221 (33%)		99 (36%)	217 (33%)	
Richest	127 (27%)	139 (30%)		75 (29%)	183 (28%)		66 (24%)	200 (30%)	
Household size [d]	4.89 (1.92)	5.12 (2.05)	0.077	4.82 (1.90)	5.08 (2.03)	0.079	4.95 (2.00)	5.03 (1.99)	0.562
Female-headed household	313 (66%)	296 (64%)	0.446	169 (66%)	426 (64%)	0.701	186 (67%)	423 (64%)	0.363
Region			<0.001			<0.001			<0.001
Iringa	185 (39%)	287 (62%)		104 (40%)	354 (53%)		92 (33%)	380 (57%)	
Mbeya	290 (61%)	179 (38%)		154 (60%)	310 (47%)		186 (67%)	283 (43%)	
**Adolescent characteristics**									
Sex			0.012			0.002			<0.001
Female	226 (48%)	184 (39%)		132 (51%)	266 (40%)		148 (53%)	262 (40%)	
Male	249 (52%)	282 (61%)		126 (49%)	398 (60%)		130 (47%)	401 (60%)	
Age in years [d]	15.91 (1.50)	16.10 (1.60)	0.053	15.88 (1.49)	16.06 (1.58)	0.105	15.87 (1.51)	16.06 (1.57)	0.092
Education/employment status			0.276			0.050			0.281
Attending school/training	249 (52%)	249 (53%)		141 (55%)	346 (52%)		140 (50%)	358 (54%)	
Engaged in paid work	45 (9%)	50 (11%)		15 (6%)	79 (12%)		24 (9%)	71 (11%)	
In school/training and paid work	22 (5%)	11 (2%)		11 (4%)	22 (3%)		13 (5%)	20 (3%)	
Not in employment, education or training	159 (33%)	156 (33%)		91 (35%)	217 (33%)		101 (36%)	214 (32%)	
Has a partner	78 (16%)	60 (13%)	0.124	43 (17%)	93 (14%)	0.306	44 (16%)	94 (14%)	0.514
Social support score, 1-5 [d,e]	4.00 (0.66)	4.03 (0.59)	0.538	3.99 (0.68)	4.02 (0.61)	0.424	3.94 (0.67)	4.05 (0.61)	0.012
**Adolescent psychosocial well-being**									
Self-esteem score, 1-5 [d,f]	3.96 (0.78)	3.97 (0.75)	0.729	3.92 (0.76)	3.98 (0.77)	0.307	3.94 (0.79)	3.97 (0.76)	0.584
Quality of life scale, 1-10 [d,g]	3.86 (2.48)	3.92 (2.48)	0.703	3.70 (2.45)	3.97 (2.48)	0.135	3.68 (2.39)	3.98 (2.51)	0.087
Locus of control score, 1-5 [d,h]	3.24 (0.48)	3.16 (0.48)	0.014	3.24 (0.49)	3.18 (0.48)	0.073	3.19 (0.47)	3.20 (0.49)	0.670
**Adolescent mental health** [i]									
Depression scale, 1-30 [d]	6.53 (4.61)	6.82 (4.91)	0.352	6.50 (4.50)	6.74 (4.85)	0.494	6.46 (4.41)	6.76 (4.90)	0.375
Exhibits depressive symptomatology	131 (28%)	143 (31%)	0.294	69 (27%)	199 (30%)	0.333	74 (27%)	200 (30%)	0.275

[a] Mentorship missing for 19 youth due to incorrect skip at start of endline data collection
[b] *P* values were derived from independent t test for continuous variables and chi square for categorical variables.
[c] Household wealth was not available for two observations
[d] Values are expressed as mean (standard deviation).
[e] Social support score is the average of four Multidimensional Scale of Perceived Social Support items.
[f] Self-esteem score is the average of two Rosenberg's self-esteem scale items.
[g] Quality of life is measured using a 10-point Cantril's Ladder of Life Scale.
[h] Locus of control score is the average of five Levenson's multidimensional locus of control scale items.
[i] Depression is measured using the 10-item Centre for Epidemiological Depression full scale as well as a binary measurement (≥10 scale score) which indicates exhibiting depressive symptomatology.

Web Table 3. Intervention effects on primary and exploratory (mediating) outcomes at endline by sex, Adolescent Cash Plus Evaluation, Tanzania, 2018–2019

Outcome	Males						Females					
	Intervention (n=941)	Control (n=992)	RD (95% CI) [a]	aOR (95% CI) [a]	*P* Value	ICC	Intervention (n=941)	Control (n=992)	RD (95% CI) [a]	aOR (95% CI) [a]	*P* Value	ICC
	N (%)	N (%)					N (%)	N (%)				
**Primary outcomes** [b]												
Depression scale, 1-30 [c]	5.83 (4.39)	6.12 (4.49)	-0.20 (-0.80, 0.40)		0.509	0.04	5.35 (4.25)	5.92 (5.10)	-0.56 (-1.21, 0.09)		0.091	0.04
Exhibits depressive symptomatology	118 (22%)	153 (29%)		0.73 (0.54, 0.98)	0.038	0.06	64 (16%)	109 (24%)		0.59 (0.41, 0.85)	0.004	0.03
**Exploratory outcomes**												
Education/employment status												
Attending school/training	152 (29%)	145 (27%)		0.96 (0.68, 1.37)	0.833	0.04	143 (35%)	183 (40%)		0.79 (0.55, 1.14)	0.208	0.00
Engaged in paid work	160 (30%)	145 (27%)		1.32 (0.98, 1.77)	0.068	0.00	59 (14%)	58 (13%)		1.21 (0.80, 1.83)	0.361	0.03
In school/training and paid work	12 (2%)	24 (4%)		0.46 (0.20, 1.04)	0.062	0.18	9 (2%)	20 (4%)		0.45 (0.18, 1.12)	0.085	0.24
Not in employment, education or training	207 (39%)	220 (41%)		0.85 (0.64, 1.13)	0.261	0.02	199 (49%)	197 (43%)		1.28 (0.94, 1.75)	0.123	0.00
Has a partner	221 (42%)	225 (42%)		1.07 (0.81, 1.42)	0.616	0.03	170 (41%)	184 (40%)		1.11 (0.81, 1.52)	0.517	0.03
Self-esteem score, 1-5 [c,d]	3.77 (0.80)	3.72 (0.77)	0.05 (-0.05, 0.15)		0.321	0.02	3.98 (0.77)	3.81 (0.86)	0.17 (0.06, 0.28)		0.003	0.00
Quality of life scale, 1-10 [c,e]	4.70 (2.14)	4.72 (2.00)	-0.06 (-0.34, 0.22)		0.674	0.04	5.03 (2.08)	4.89 (1.98)	0.14 (-0.16, 0.44)		0.369	0.08
Locus of control score, 1-5 [c,f]	3.25 (0.46)	3.28 (0.46)	-0.03 (-0.08, 0.03)		0.315	0.02	3.35 (0.45)	3.28 (0.48)	0.06 (0.00, 0.13)		0.043	0.05

Abbreviations: aOR, adjusted odds ratio; CI, confidence interval; ICC, intraclass correlation coefficient; RD, risk difference.

[a] Adjusted for age in years, the corresponding outcome at baseline, and district/community size fixed effects (the strata). Multilevel methodology was used to account for clustering of outcome within and between villages. Robust 95% CIs in parentheses.

[b] Primary outcomes are measured using the 10-item Center for Epidemiologic Studies Depression Scale as well as a binary measurement (≥10 scale score) which indicates exhibiting depressive symptomatology.

[c] Values are expressed as mean (standard deviation).

[d] Self-esteem score is the average of two Rosenberg's self-esteem scale items.

[e] Quality of life is measured using a 10-point Cantril's Ladder of Life Scale.

[f] Locus of control score is the average of five Levenson's multidimensional locus of control scale items.
